# Sclerosing mesenteritis as a rare cause of abdominal pain and intraabdominal mass: a cases report and review of the literature

**DOI:** 10.1186/1757-1626-1-242

**Published:** 2008-10-16

**Authors:** Guo-Li Gu, Shi-Lin Wang, Xue-Ming Wei, Li Ren, De-Chang Li, Fu-Xian Zou

**Affiliations:** 1 Department of General Surgery, the General Hospital of Chinese PLA Air force, Beijing 100142, PR China; 2 Department of Pathology, the General Hospital of Chinese PLA Air force, Beijing 100142, PR China; 3 Surgery of the Affiliated Hospital, Jiangxi University of Science and Technology, Ganzhou City, Jiangxi Province 341000, PR China

## Abstract

Sclerosing mesenteritis is a rare, benign, and chronic fibrosing inflammation disease with unknown etiology that affects the mesentery of small bowel and colon. The disease has two well-established histological types: the acute or subacute form known as mesenteric panniculitis and the chronic form known as retractile or sclerosing mesenteritis. Because the sclerosing mesenteritis is lack of special clinical manifestation and typical signs, so the patients are very easy to be misdiagnosed. The correct diagnosis of sclerosing mesenteritis depends on pathological examination and exploratory laparotomy. We report a case of sclerosing mesenteritis in a 52-year-old male who presented with chronic abdominal pain and intraabdominal mass. This patient had a long-term and heavy drinking history. He was misdiagnosed as celiac teratoma by CT examination and then underwent an exploratory laparotomy at March 2 2004. A mass, its diameter being about 5 cm, was detected in mesentery of distal ileum. Although a few small intestines tightly adhered on the mass, the involved intestine had no obstruction. The intraoperative biopsy indicated that it was an inflammatory mass. The mass and adhered intestines were removed. He was diagnosed with sclerosing mesenteritis by histopathological examination of paraffin section. After operation, this patient went well and lives without recrudescence at the time we wrote this paper.

## Introduction

Sclerosing mesenteritis is a rare, benign, and chronic fibrosing inflammation disease with unknown etiology that affects the mesentery. On rare occasions, it may involve the mesocolon, peripancreatic region, omentum, retroperitoneum, or pelvis [1]. Sclerosing mesenteritis can be categorized into three pathological changes [2]: chronic non-specific inflammation, fat necrosis and fibrosis. Based on the different course of disease, many terms have been used to describe sclerosing mesenteritis [3], including mesenteric lipodystrophia, retractile or liposclerotic mesenteritis, mesenteric Weber-Christian disease, xantogranulomatous mesenteritis, mesenteric lipogranuloma, and systemic nodular panniculitis. This varied terminology has caused considerable confusion, but the condition can now be evaluated as a single disease with two pathological subgroups. If inflammation and fat necrosis predominate over fibrosis, the process is known as mesenteric panniculitis; when fibrosis and retraction predominate, the result is retractile mesenteritis. The overall presence of some degree of fibrosis makes the pathologic term *sclerosing mesenteritis *more accurate in most cases [3].

In most patients with sclerosing mesenteritis, the condition consists of a mixture of chronic non-specific inflammation, fat necrosis and fibrosis. So their clinical manifestation can vary. Patients may present with abdominal pain, intestinal obstruction, fever, chylous ascites, a mass, constipation or diarrhea [4,5]. Because its clinical manifestations are nonspecial and atypical, so the preoperative diagnosis of sclerosing mesenteritis can be very difficult [6]. Clinically, it is a grave challenge for diagnosis to radiologist, surgeon and even pathologist facing with such patients in clinical work. In order to avoid misdiagnosis; the surgeon, radiologist and pathologist should keep astute clinical suspicion about it. We report a rare case of a 52-year-old male patient with sclerosing mesenteritis who presented with chronic abdominal pain and intraabdominal mass. He was misdiagnosed as celiac teratoma by computed tomography (CT) scan and then underwent exploratory laparotomy and partial enterectomy. At last, he was diagnosed correctly by histopathological examination of paraffin section.

## Case presentation

**Figure 1:**
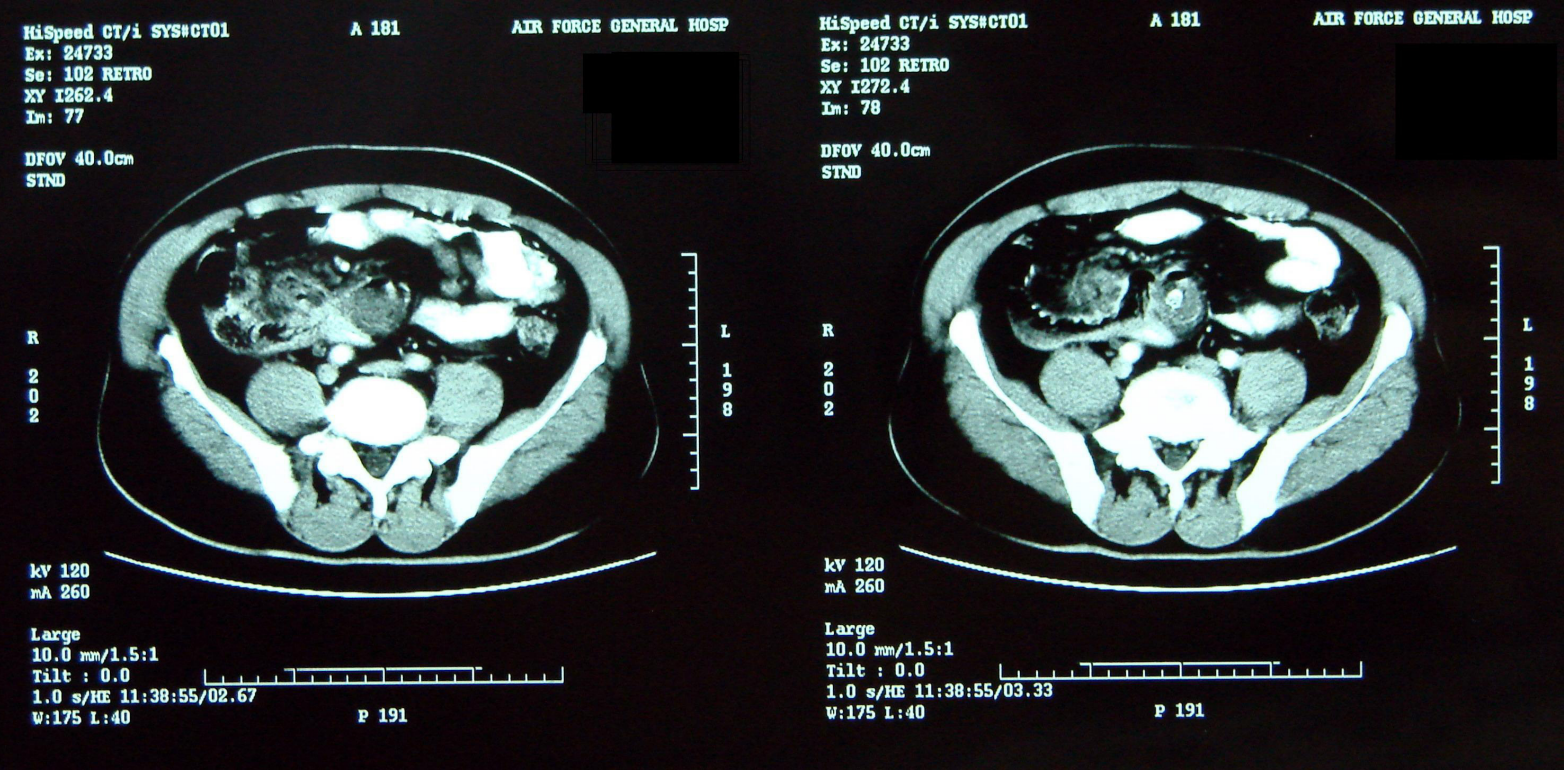
**Abdominal CT scan**. Sclerosing mesenteritis in a 52-year-old male with a long history of abdominal pain. Axial CT scan demonstrates a solid soft-tissue mass with calcification in the right lower abdomen, which was related to small bowel and mesentery. The mass had a typical "fat ring sign" and a calcified core.

**Figure 2:**
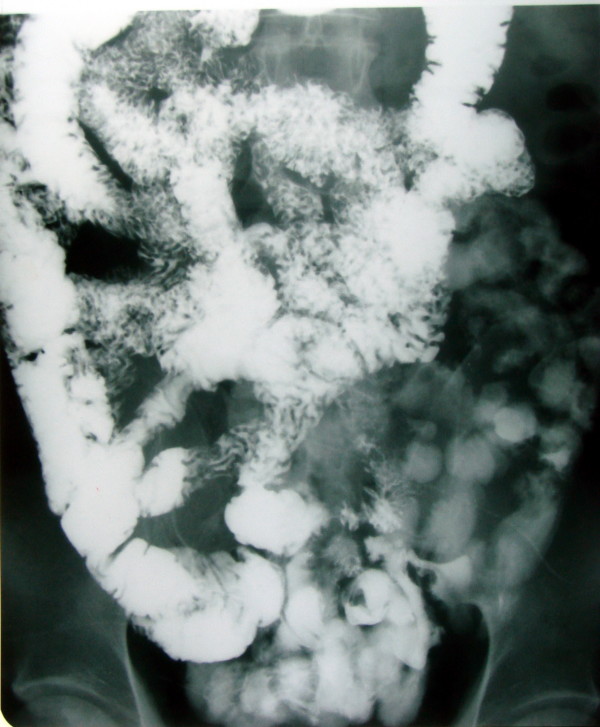
**Gastrointestinal barium meal examination**. Gastrointestinal barium meal examination indicates that distal ileum is tangled, however, without obstruction.

**Figure 3:**
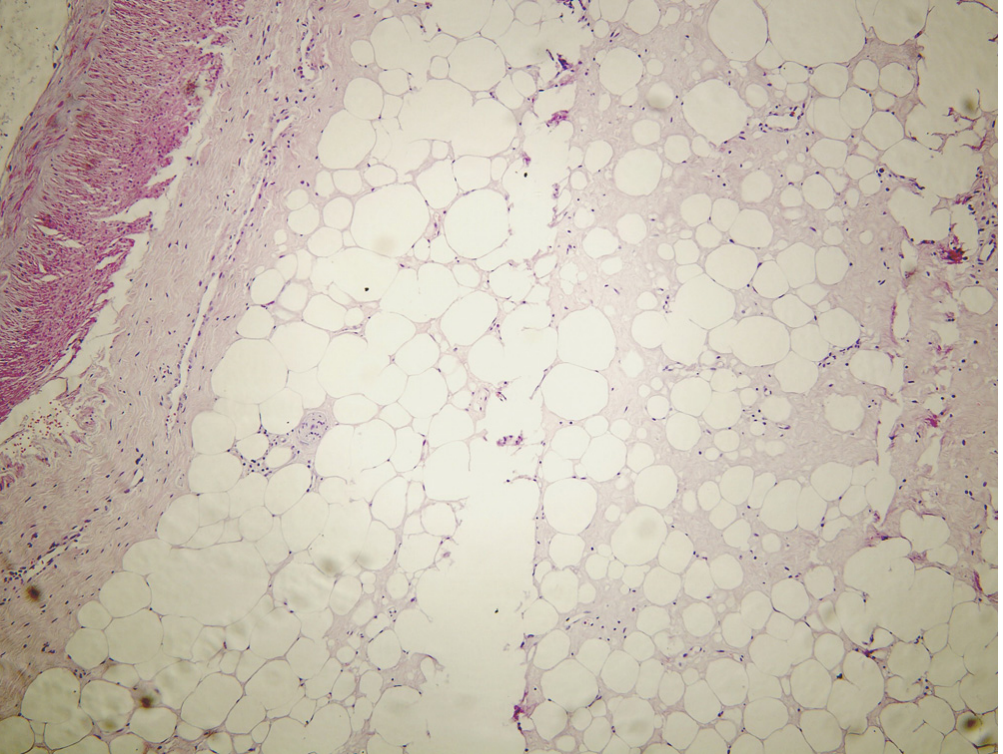
**Photomicrograph 1 (original magnification, ×200; HE stain)**. Photomicrograph 1 shows fat necrosis, sclerosing fibrosis in mesentery mass, and the inflammatory stops abruptly at the edge of bowel wall.

**Figure 4:**
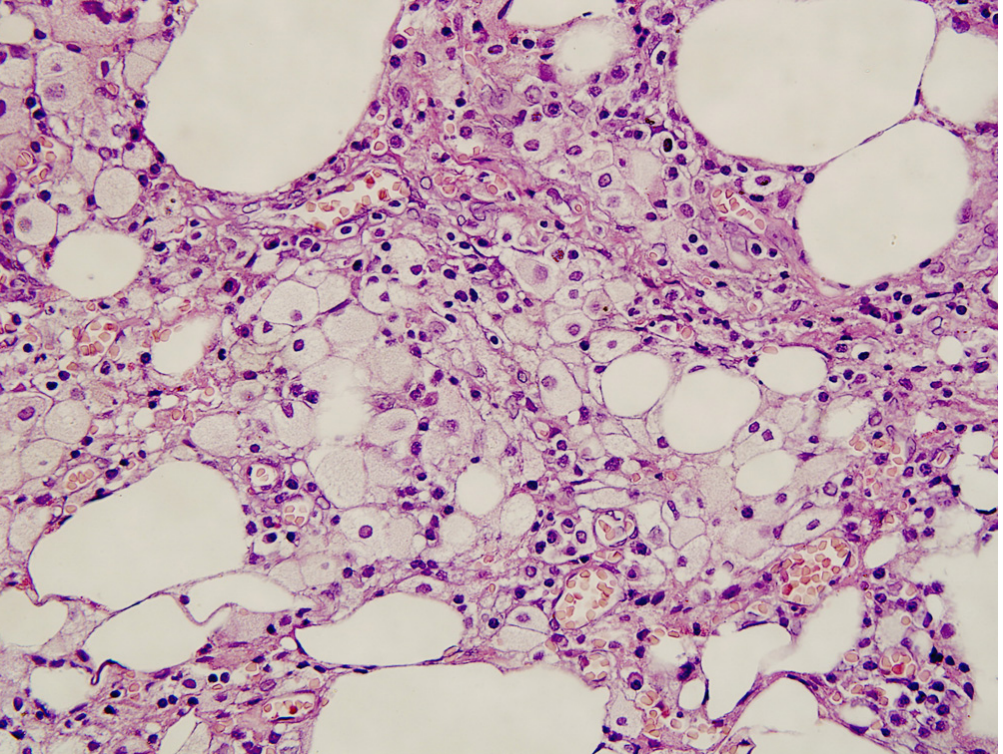
**Photomicrograph 2(original magnification, ×400; HE stain)**. In this view of fat necrosis at high magnification, Photomicrograph 2 shows clusters of inflammatory cells and lipid-laden macrophages are seen between the necrotic adipose tissue cells in mesentery mass.

A 52-year-old male patient presented with chronic abdominal pain for about 6 mo and intraabdominal mass for about 1 mo. The abdominal pain, mainly located around navel, was intermittent and mild. The mass, located in the right lower quadrant, was mobile, smooth, rigid and about the size of a fist. The laboratory profile of routine blood test, renal and hepatic function tests were normal. Abdominal CT scan demonstrated a solid soft-tissue mass with calcification in the right lower abdomen, which correlated with small bowel and mesentery (Figure 1). Barium meal examination indicated that distal ileum was tangled without obstruction (Figure 2). For short of experience, the radiologist misdiagnosed this patient with celiac teratoma. So the patient underwent an exploratory laparotomy in our department at March 2 2004. A mass, its diameter being about 5 cm, was detected in mesentery of distal ileum. A few ileums tightly adhered on the mass and showed chronic ischemic condition with scars on the serosal surface; however the involved intestine had no obstruction. The three times intraoperative frozen section indicated that it was an inflammatory mass. The mass and adhered intestines were removed. The biopsy of pathological specimens with paraffin section showed fat necrosis, sclerosing fibrosis, clusters of inflammatory cells and lipid-laden macrophages in mesentery mass; and the inflammatory stopped abruptly at the edge of bowel wall (Figure 3, 4). So this case was diagnosed with sclerosing mesenteritis at last. The patient did not take immuno-suppressor and recovered well after operation without any digestive discomfort. No recurrence of the sclerosing mesenteritis was observed during 4 years of follow-up.

## Discussion

Sclerosing mesenteritis is a rare disease of unknown etiology that is characterized by tumor-like mass composed of chronic nonspecific inflammation, fat necrosis and fibrosis [1-4,7]. Chronic bellyache and intraabdominal mass are its main clinical manifestations [1,2,7]. Although various causes have been suggested, including infection, trauma or ischaemia of the mesentery, self-immune; the exact etiology of the disease still cannot affirm [8]. In most cases, sclerosing mesenteritis involves the alvine mesentery, but it can also affect the mesocolon, peripancreatic region, omentum, retroperitoneum, or pelvis [1-3,5]. Sclerosing mesenteritis is reportedly relevant to other pathological processes such as vasculitis, granulomatous disease, pancreatitis and malignancy [9].

Even until now, just about 300 cases have been reported in the world literatures [10]. According to literatures saying, few or none of the patients with sclerosing mesenteritis can be correctly diagnosed before operation [3-10]. Although a few scholars [11,12] said that abdominal CT scan and magnetic resonance imaging (MRI) take important roles in suggesting the accurate diagnosis and can be used for distinguishing sclerosing mesenteritis from other mesenteric diseases with similar imaging features such as carcinomatosis, carcinoid tumor, lymphoma, desmoid tumor, and mesenteric edema. The imaging appearances of sclerosing mesenteritis vary depending on the predominant tissue component (fat necrosis, inflammation, or fibrosis) [13]. So the accurate diagnosis of sclerosing mesenteritis can be established only by evaluating a biopsy specimen. Because sclerosing mesenteritis is very rare and lack of special clinical manifestation and typical signs; so it is very easy to be misdiagnosed not only by radiologist and surgeon, but also by pathologist. As to this case, he was misdiagnosed as celiac teratoma by radiologist and then underwent an exploratory laparotomy. According as the mass and affected bowel, we misdiagnosed this patient as intestinal cancer or gastrointestinal stromal tumors (GIST) during operation and removed the mass and affected bowel; although three times intraoperative frozen section indicated that it was an inflammatory mass. The pathologist only diagnosed it as inflammatory mass in intraoperative biopsy, not sclerosing mesenteritis. At last, this patient was diagnosed via the consultation of paraffin section. We think the chief reason of misdiagnosis during preoperation and operation is that we lack experience on sclerosing mesenteritis and do not keep astute clinical suspicion about it. In summary, it is a serious challenge to diagnose this unspecific, benign inflammatory disease; both for the radiologist, surgeon and pathologist.

At the present time, the scholars still have not reached a consensus on the treatment of sclerosing mesenteritis [1-8]. Some authors recommend that asymptomatic or mild symptoms sclerosing mesenteritis may be left untreated and observed, surgical resection is advocated for patients with life-threatening complications such as bowel obstruction or perforation. Others recommend surgical resection or aggressive immunosuppressive therapy with prednisone and thalidomide to prevent progression of the lesion once the diagnosis is established. We think the therapeutical nodus of sclerosing mesenteritis still consists in its diagnosis. The tumor-like mass of sclerosing mesenteritis is very difficult to distinguish from other mesenteric diseases and bowel tumor during preoperation and operation. In this case, although three consecutive frozen sections indicated that it was an inflammatory mass; because of worrying about the inaccuracy of frozen section, we had to remove the tumor-like mass and involved bowels in order to avoid repetitive operation. Because the mass consist of lots of fat, so the clinical significance of frozen section in diagnosing sclerosing mesenteritis is finite. The surgeons, like us, usually have to resect the focus to avoid reoperation, although the focuses do not need excision at all in most cases.

Literatures revealed that sclerosing mesenteritis have a favorable prognosis and may be self-limiting [1-4,14]. There is a long-running debate as to whether immunosuppressant drug should be used in treatment of sclerosing mesenteritis [1-6,13]. In this case, he did not take immunosuppressant and recovered well after operation without any digestive discomfort. No recurrence of the sclerosing mesenteritis was observed at the time when we wrote this report. From the literatures experiences [1-3,13], it need more research to study this subject. We were released to see that few scholars had made an attempt and obtained fine effect [14].

## Competing interests

The authors declare that they have no competing interests. We have obtained consent for publication in print and electronically from the patient. We all authors have seen and approved the submitted version of this manuscript. There was not a medical writer or editor involved in the generation of our manuscript. We are assured that the manuscript has not been published or submitted for publication elsewhere except as a brief abstract in the proceedings of a scientific meeting or symposium.

## Authors' contributions

GG, WSL and WXM contributed equally to this work; GGL and WSL designed the research; GGL and ZFX collected the clinical data and wrote the manuscript; RL and LDC collected the pathological data; WSL, WXM and ZFX revised the manuscript.

## Consent

The consent was obtained from the patient for publication of this case report and accompanying images.
